# Comparing RIEGL RiCOPTER UAV LiDAR Derived Canopy Height and DBH with Terrestrial LiDAR

**DOI:** 10.3390/s17102371

**Published:** 2017-10-17

**Authors:** Benjamin Brede, Alvaro Lau, Harm M. Bartholomeus, Lammert Kooistra

**Affiliations:** 1 Laboratory of Geo-Information Science and Remote Sensing, Wageningen University & Research, Droevendaalsesteeg, 36708 PB Wageningen, The Netherlands; alvaro.lausarmiento@wur.nl (A.L.); harm.bartholomeus@wur.nl (H.M.B.); lammert.kooistra@wur.nl (L.K.); 2 Center for International Forestry Research (CIFOR), Situ Gede, Sindang Barang, Bogor 16680, Indonesia

**Keywords:** UAV, LiDAR, ALS, TLS, forest inventory

## Abstract

In recent years, LIght Detection And Ranging (LiDAR) and especially Terrestrial Laser Scanning (TLS) systems have shown the potential to revolutionise forest structural characterisation by providing unprecedented 3D data. However, manned Airborne Laser Scanning (ALS) requires costly campaigns and produces relatively low point density, while TLS is labour intense and time demanding. Unmanned Aerial Vehicle (UAV)-borne laser scanning can be the way in between. In this study, we present first results and experiences with the RIEGL RiCOPTER with VUX${}^{®}$-1UAV ALS system and compare it with the well tested RIEGL VZ-400 TLS system. We scanned the same forest plots with both systems over the course of two days. We derived Digital Terrain Models (DTMs), Digital Surface Models (DSMs) and finally Canopy Height Models (CHMs) from the resulting point clouds. ALS CHMs were on average 11.5 cm higher in five plots with different canopy conditions. This showed that TLS could not always detect the top of canopy. Moreover, we extracted trunk segments of 58 trees for ALS and TLS simultaneously, of which 39 could be used to model Diameter at Breast Height (DBH). ALS DBH showed a high agreement with TLS DBH with a correlation coefficient of 0.98 and root mean square error of 4.24 cm. We conclude that RiCOPTER has the potential to perform comparable to TLS for estimating forest canopy height and DBH under the studied forest conditions. Further research should be directed to testing UAV-borne LiDAR for explicit 3D modelling of whole trees to estimate tree volume and subsequently Above-Ground Biomass (AGB).

## 1. Introduction

LIght Detection And Ranging (LiDAR) has become a valuable source of information to assess vegetation canopy structure. This is especially true for complex forest canopies that limit manual and destructive sampling. These capabilities are investigated to replace traditional forest plot inventories [1], but even more if they can deliver additional information that is not captured with traditional inventories [2]. One particular important variable in this context is Above-Ground Biomass(AGB) which makes up an essential part of the forest carbon pool. Terrestrial Laser Scanning (TLS) has the potential to accurately measure AGB on a plot scale [3,4], while Airborne Laser Scanning (ALS) from manned aircraft can serve as means to up-scale plot measurements to the landscape level. This is particularly interesting for calibration and validation activities of space-borne missions aiming at AGB assessment like ESA’s BIOMASS [5] and NASA’s GEDI (https://science.nasa.gov/missions/gedi) missions. Another important derivative of LiDAR point clouds is vertical forest canopy structure, which is linked to biodiversity [6,7].

ALS is typically acquired from manned aircraft, thereby covering large areas, but requiring substantial financial capital and available infrastructure. Acquisition density is typically in the order of 1 to 10 points/$m^{2}$, depending on flight altitude and scanner configuration. A straight-forward application for ALS point clouds is the generation of Digital Terrain Models (DTMs) and Digital Surface Models (DSMs), and derivation of canopy height by considering the difference between those two. More advanced products take into account the waveform of the returning pulses and reconstruct canopy attributes from that [8]. However, the relatively low density of ALS point clouds forces to approach actual canopy structure from a statistical point of view where each resolution cell contains a sample of the population of possible returns. In this respect, ALS products can be treated as 2.5D raster layers.

On the other hand, TLS produces point clouds with such a density—millions of points per scan position—that single canopy elements like stems and branches can be resolved. Geometrical models serve to reconstruct the 3D tree architecture, and allow estimation of wood volume and derivation of AGB [4,9,10] and other stand characteristics. A hard requirement for this approach is accurate co-registration of several point clouds acquired from different scan positions in the forest, which leads to time demanding field campaigns, mostly in the order of 3 to 6 days/ha [11]. Therefore, it is questionable if TLS in its current form will replace operational plot inventories, or rather supply higher quality information for selected samples [2].

Independent from the developments of LiDAR instruments, Unmanned Aerial Vehicles (UAVs) have found use as platforms for various types of sensors in forestry and many other fields [12,13]. Especially the introduction of affordable, ready-to-use systems on the consumer market has been boosting applications and widened the user community. Even consumer-grade RGB cameras in combination with dedicated software packages can serve for the production of high-resolution orthomosaics and surface models derived with Structure from Motion (SfM) techniques. More sophisticated prototype sensors also allow the production of hyperspectral images [14]. One of the most favourable aspects of UAVs as sensor platforms is their low demand in infrastructure, high mapping speed and price advantage compared to manned aircraft. The implementation of legal regulations for professional UAV users remains a hot topic however [12].

Recently, LiDAR sensors have been mounted on UAVs to combine the advantages of LiDAR and UAV technology. A variety of custom build systems with different degrees of off-the-shelf components were tested to derive forest inventory metrics. Jaakkola et al. [15] probably build the first UAV LiDAR system, the Finish Geodetic Institute (FGI) Sensei, integrating an Ibeo Lux and Sick LMS151 profile scanner. During test flights the Sensei produced point clouds with 100 to 1500 points/$m^{2}$ and could be successfully used to detect single trees. Another custom build system based on the Ibeo Lux scanner was presented by Wallace et al. [16]. During tests it produced point clouds with up to 50 $\mathrm{points}/m^{2}$, but with a relatively low airborne time of 3 to 5 min owed to the capacity of the UAV. This same system was employed to conduct surveys of forest plots, and terrain and under-storey height, tree location, tree height, crown area and volume could be derived [17]. Chisholm et al. [18] constructed another light-weight LiDAR UAV system that did not require any means of positioning or orientation system, but rather used pattern-matching algorithms to produce a point cloud. However, due to assumptions in the processing the system and the low range of the laser scanner of 30 m had to be flown below canopy. They could successfully estimate Diameter at Breast Height (DBH) for their open grove study site. Wei et al. [19] employed the commercially available HawkScan1200, consisting of a VUX${}^{®}$-1LR scanner and Applanix AP20 Inertial Measurement Unit (IMU), and mapped a 60 ${\mathrm{km}}^{2}$ area with a point density of 0.5 $\mathrm{points}/m^{2}$ to perform vegetation filtering and DTM generation on the resulting point cloud.

Overall, these systems showcase that principal technological challenges such as component miniaturisation and suitable post-processing have been overcome in the recent years. Important forest inventory metrics like tree height, location and DBH could be derived. Nonetheless, custom-build systems have not yet achieved point density counts in same the order of magnitude as TLS. This would open up opportunities that are at the forefront of LiDAR research in forestry, such as explicit structural modelling to precisely estimate AGB [4,9]. Moreover, even though custom build systems are low cost, at the same time they are typically not easily available for use by a wider audience.

The aim of this paper is to present the commercially available RIEGL RiCOPTER system and the work flow to process the acquired data. In a field experiment we concurrently collected RiCOPTER and TLS data in a forest site containing different canopy architectures. We compared the two point clouds in respect to their point distributions, different elevation models derived from both point clouds and estimates of DBH. With this comparison we want to test if the RiCOPTER performs comparable to TLS field acquisition.

## 2. RIEGL RiCOPTER with VUX${}^{®}$-1UAV

### 2.1. System Specifications

The RIEGL RiCOPTER with VUX${}^{®}$-1UAV (RIEGL Laser Measurement Systems GmbH, Horn, Austria) is an integrated UAV and sensor system. The RiCOPTER is a battery-driven octocopter with an empty weight (no batteries and equipment) of 9 kg that can carry a payload of up to 8 kg. For safety reasons it has two flight controller units. In case of system failure of the main controller, the backup takes over immediately. Together with the VUX${}^{®}$-1UAV scanner (3.75 kg), the system controller (0.9 kg), the IMU (0.7 kg) and optional cameras the total system weights just under 25 kg; hence, it is possible to operate it under light UAV regulations in many European countries [13]. The batteries allow flight times of up to 30 min at 30 km h${}^{-1}$ maximum cruise speed. This allows flying multiple overlapping flight lines to increase target coverage. However, during mapping of forest plots flight time and speed need to be reduced to guarantee flight safety and adequate point density.

The VUX${}^{®}$-1UAV is a survey-grade laser scanner that is mounted underneath the RiCOPTER. It uses a rotating mirror with a rotation axis in flight direction to direct the laser pulses and achieve an across-track Field Of View (FOV) of 330° perpendicular to the flight direction. This means that lateral flight line overlap is only restricted by the maximum operating range of the laser. Overall its attributes are comparable to the terrestrial VZ-400 despite its lower weight (Table 1). It should be noted that both operate at a wavelength of 1550 nm, which makes them eye-safe and sensitive to the same types of canopy elements. An Applanix AP20 IMU attached to the VUX${}^{®}$-1UAV and Global Navigation Satellite System (GNSS) antennas on top of the RiCOPTER record flight orientation and GNSS data. Apart from these devices and sensors essential for processing, two consumer-grade Sony Alpha-6000 system cameras can be mounted on the VUX${}^{®}$-1UAV. During later processing the point clouds can be overlaid with the RGB colour information from these cameras. The on-board instrument controller manages all sensors’ data streams and includes a 220 GB SSD storage, which is sufficient for several missions.

Next to the RiCOPTER system a ground station is necessary for mission planning and in-flight coordination. Planar or spherical Ground Control Point (GCPs) should be set out in the field before flight to support co-registration during processing. These targets do not necessarily need to be geolocated in case only internal point cloud registration is to be optimised. However, they should have an adequate size of >0.5 m—depending on flight altitude and scanning speed—to be properly covered. In case sufficient planar surfaces are available in the study area, these can also be used. However, this is typically not the case for forest plots.

### 2.2. Operations

Necessary legal requirements for professional operations are similar to other UAV operations and mainly involve RiCOPTER registration as an aircraft in the country of operations as well as the training and licensing of the pilot. Both processes can partly run in parallel and can take up to several months. Additional to regular licensing the pilot should also become familiar with the flight behaviour of the RiCOPTER, since it is considerably larger than typical mini-UAV. Also the proper operation of the two independent flight controllers needs to be trained. Moreover, operation in forest areas usually requires take off and landing in canopy openings with restricted viewing conditions and options to manoeuvre. Another general preparation includes the identification of a source of base station data that is necessary for processing the acquired data. Additionally, legal requirements for the transportation of the batteries need to be investigated.

Once these general prerequisites are fulfilled, practical mission planning can begin. This mainly involves getting access permissions to the study site especially from the landowner, arranging transport and notifying other airspace users. Furthermore, the weather forecast should be studied with respect to wind, visibility and humidity to identify the best suitable days for mission execution. As for other mini-UAV the RiCOPTER has a legal limit on wind speed up to which take off is allowed, which is 7 $m\;s^{-1}$ for the Netherlands. However, wind limits are typically stricter in respect to data quality as crown movement hampers proper co-registration of point clouds from different flight lines, as is also the case for TLS [11].

Initial flight path planning should be performed in preparation of the field work. The target is a certain point density to be achieved by varying flying speed and altitude, and overlap of flight lines. Nonetheless, not anticipated on-site conditions like single emerging trees or lack of emergency landing locations can demand modification. Transport to the site should take into account the size and weight of the equipment. The RiCOPTER itself is delivered with a transport case of ~120 cm × 80 cm × 55 cm. The ground station has dimensions ~55 cm × 45 cm × 20 cm. At the study site, the area should be inspected to identify take-off and landing as well as emergency landing locations, obstacles close the intended flight path and positions for GCPs. After completion the equipment can be set up and the mission executed. After the mission, the raw data is downloaded from the instrument controller.

### 2.3. Data Processing

**Figure 1:**
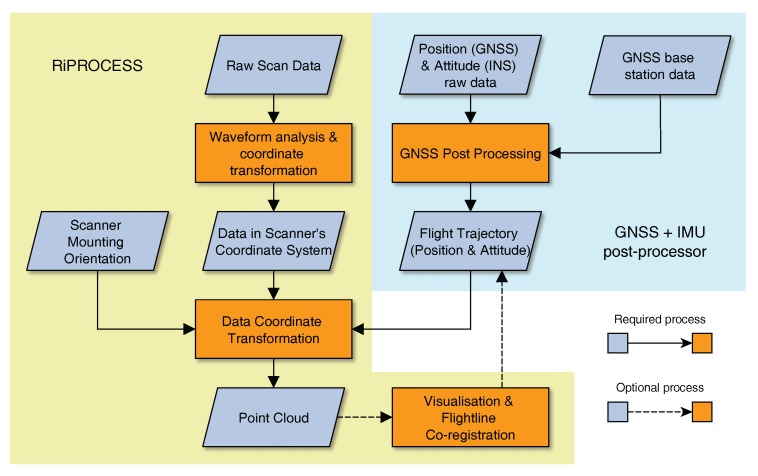
RiCOPTER processing flowchart (based on [20]).

RIEGL provides a software suite together with the RiCOPTER system to convert the produced raw data into point clouds. Figure 1 gives an overview of the required steps. While most of the work can be done in RIEGL’s software for airborne and mobile laser scanning, RiPROCESS, the trajectory preprocessing has to be accomplished with third party software, e.g., Applanix POSPac Mobile Mapping Suite. For this purpose additional GNSS base station data has to be acquired. During GNSS post-processing both data streams from the GNSS antennas and the IMU are taken into account to reconstruct the flight trajectory.

For each flown and logged scan line, the raw scan data has to be subjected to waveform analysis during which targets are detected within the stored flight line waveforms. Up to four targets can be detected per pulse. During this process, Multiple Time Around (MTA) range ambiguities have to be taken care of. MTA range ambiguity occurs when pulses are fired before their predecessor pulses can return. MTA 1 range, where no ambiguity can occur because a pulse always returns before the next is fired, is at around 100 m range for a Pulse Repition Rate (PRR) of 550 Hz. Thus MTA range ambiguity needs to be taken care of, but does not result in serious obstacles assuming flying heights below 120 m. The waveform processing detects targets in the scanners own coordinate system. Next, this data is interpreted with help of the trajectory information and the scanner mounting orientation to produce the first point clouds, one per flight line. The within and across flight line registration is already of high quality as experienced by the authors during several missions and would serve when registration error of below 1 m is not an issue.

However, when sub-centimetre accuracy is required, point clouds need to be fine registered. In principal this means to reduce errors in the flight trajectory as the position and orientation error of the RiCOPTER system (cm scale) is much bigger than the range error of the scanner (mm scale). This process can include two independent steps. One is the within flight line trajectory optimisation that is handled by the RiPRECISION package within RiPROCESS. Similar to Multi-Station Adjustment (MSA) for TLS [11] control planes are automatically searched for in the point clouds per flight line. So far this process demands no user interaction. It can be supported by GCPs that have been independently located to within millimetres, e.g., with Real Time Kinematic (RTK) GNSS. However, for forest situations this is impractical as GNSS reception is typically too low under closed canopies to achieve the required accuracy. The other possible optimisation is across flight line optimisation that can be performed with the Scan Data Adjustment (SDA) package within RiPROCESS. It assumes within flight line registration as optimal and only changes the overall position and orientation of flight lines to each other. This can be compared to linking point clouds from single scanning positions in TLS. Here, next to automatically detected planes, also manually digitised control objects, such as planes, spheres and points, can be included to improve the co-registration.

The point cloud processing can be finished off with removal of atmospheric noise, which is visible as returns close to the flight trajectory with typically low reflectivity, and target type classification. Finished point clouds can be exported from RiPROCESS in common file formats, e.g., ASCII and LAS, to continue analysis in dedicated software packages.

## 3. Field Experiment

**Figure 2:**
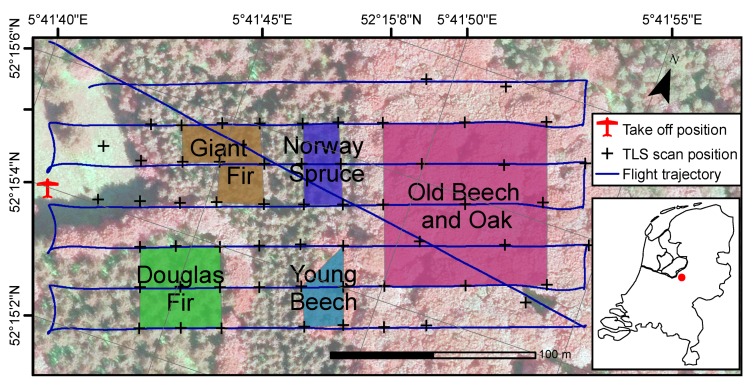
Map of the study site with TLS scan positions (crosses), take off position (red plane), flight lines (blue), target areas, study site location in the Netherlands (red dot in inset), airborne false colour composite as background image.

The field experiment took place at the Speulderbos Fiducial Reference site in the Veluwe forest area (N52°15.15′ E5°42.00′), The Netherlands [21] (www.wur.eu/fbprv). The core site is established in a stand composed of European Beech (*Fagus sylvatica*) with occasional Pedunculate Oak (*Quercus robur*) and Sessile Oak (*Quercus petraea*), and a very open understorey with only few European Holly (*Ilex aquifolium*) (Figure 2). The stand was established in 1835 and had a tree density of 204 trees/ha. At the time of the experiment the overstorey was in the progress of spring bud break and leaf unfolding, so that only few trees carried a full leaf canopy. In an earlier inventory campaign the Beech stand has been equipped with a 40 m spaced wooden pole grid that has also been geo-located with RTK GPS and land surveying techniques to an accuracy of better than 0.5 m.

Additional to the Beech stand, sections of Norway Spruce (*Picea abies*), Giant Fir (*Abies grandis*), young beech and Douglas Fir (*Pseudotsuga menziesii*) have been scanned as well with the goal to capture different forest types in terms of species composition, tree density and canopy architecture. The Norway Spruce and Giant Fir stands were established in 1943 and 1967, respectively, and had no understorey species. However, the plots were relatively dense with 676 Trees/ha and 961 Trees/ha, respectively, and many low branches. The young beech stand was established in 1973 and had a density of 805 Trees/ha. There were no other species present in this stand, but most lower branches were carrying leaves. The Douglas Fir stand had a very open understorey where only few saplings of up to 2 m height could be found. The stand was established in 1959 and has been thinned since then as was obvious through the present stumps.

The total scanned area covered 100 m × 180 m, roughly 2 ha. In the study area, a forest road separates the old beech and oak from the other stands, and a bike path the Giant Fir and Norway Spruce stands. The UAV take-off area was located in an opening east of the stands that was wide enough to allow Visual Line of Sight (VLOS) operations.

TLS data acquisition was completed in the course of two days that were both marked by very low wind speeds (<3 $m\;s^{-1}$). During the first day the TLS scan position grid was set up. For that the wooden poles in the Beech stand were taken as starting positions. With the help of a theodolite the TLS positions were marked to form a grid of 40 m spacing in the Beech and 20 m spacing in the Douglas Fir stands. Additional positions in the grid centres have been added in the Beech stand. Cylindrical retro-reflective targets were set up for later coarse co-registration of scans [11]. The first 15 positions have been scanned during the first day, the remaining 43 during the second day. All scans were performed with 0.06° scan resolution. Due to the VZ-400’s zenithal scan angle range of 30° to 130°, an upward and tilted scan had to be performed per scan location to cover the area directly over the scan position.

To support co-registration of RiCOPTER flight lines 4 large (120 cm × 60 cm) and 8 small (60 cm × 60 cm) ground control panels have been distributed under the trees and next to the take-off site. The panels consist each of 2 equally sized wooden panes connected via piano hinges. When set up the panes form a 90° angle between them, which makes them look like tents. Cars used for transport were deliberately parked on the forest road in the scanning area to provide additional control surfaces for the co-registration. The ground station was erected next to the take off site. The scan lines were designed to maximise lateral overlap and efficiently use air time (Figure 2). The RiCOPTER was flown at an altitude of 90 m a.g.l., with a cruise speed of 6 $m\;s^{-1}$. The VUX${}^{®}$-1UAV was operated with the full FOV of 330°, a PRR of 550 kHz and scan speed of 58 lines/s, which resulted in an average rectangular point spacing of ~8 cm and a point density of 140 $\mathrm{points}/m^{2}$ for a single flight line at nadir. Mission time for active scan and non-active connection lines was 9 min to cover a distance of ~2300 m.

## 4. Methods

In case of TLS, the scans were first coarsely co-registered with automatically extracted tie-points based on the retro-reflective cylinders. These registrations had typical registration errors of <5 cm. Afterwards MSA was applied to refine the registration [11]. This approach automatically searches for tie-planes in the scans and iteratively adjusts orientation and position of each scan position to minimise the global fitting error of tie-planes. The resulting standard deviation of the errors over all tie-planes was 0.62 cm. All operations were executed with RIEGL’s RiSCAN PRO${}^{®}$ software package.

For processing of the RiCOPTER data the work-flow as described in Section 2.3 was applied. GNSS data was obtained from 06-GPS (Sliedrecht, The Netherlands) for a virtual base station in the centre of the study site and the period of the campaign to allow GNSS post-processing. RiPRECSION-UAV was applied to optimise within flight line registration. Automatic across-flight line registration with automatic search of tie-planes continuously failed to produce good results, probably due to missing planar surfaces in the study area. Therefore, the GCP panels were manually digitised as tie-planes and used for fine registration. Final standard deviation of the fitting errors of 0.97 cm.

The resulting point clouds from TLS and ALS were co-registered via common tie-planes. These were the manually selected GCP panels. Then different raster products were produced at 0.5 m resolution with LAStools (https://rapidlasso.com/lastools/): scan density by counting all hits within a resolution cell, DTMs by selecting the lowest point in a resolution cell, DSMs with the highest, and Canopy Height Models (CHMs) by calculating the difference between DTMs and DSMs.

The lower stem parts of individual trees were manually extracted from the TLS and ALS point clouds from the 5 plots (Figure 2). For each tree all points at a height of 120 to 140 cm were selected to represent DBH. These subsets were manually inspected for the suitability to fit circles. In case of presence of branches at the DBH height, the corresponding points were further manually removed. Next, circles were fitted to the horizontal coordinates of these points separately for ALS and TLS. An iterative optimisation procedure was used to minimise the euclidean distance between points and circles according to Coope [22] as implemented in R’s (http://www.r-project.org/) circular package. Next to the geometries, the points contained information about the return number and scan angle under which they were recorded. These were analysed to gain more insights which scan conditions can be beneficial to record stem points.

## 5. Results

**Figure 3:**
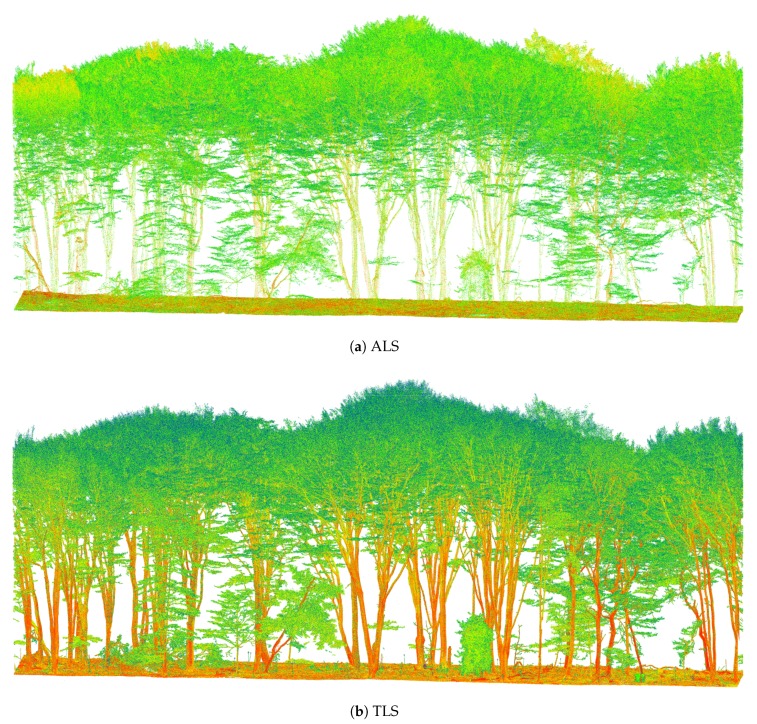
Old Beech and Oak point clouds samples from same perspective, coloured according to apparent reflectance (blue = low, green = medium, red high).

The acquired TLS and ALS point clouds showed a number of differences. Figure 3 shows two sample transects through the Old Beech and Oak area (cf. Figure 2). The ALS point cloud clearly had a lower point density in the stem area, while the branch and leaf area appeared to be of comparable density. Nonetheless, single stems as well as larger branches could be made out. It should be noted that even though the images suggest the trees to have a full canopy, this was not the case during the time of acquisition. The canopy level rather has a distinctively different apparent reflectance than stem and ground elements, because partial hits are much more likely in the crown area where branches do not completely fill the laser footprint.

**Figure 4:**
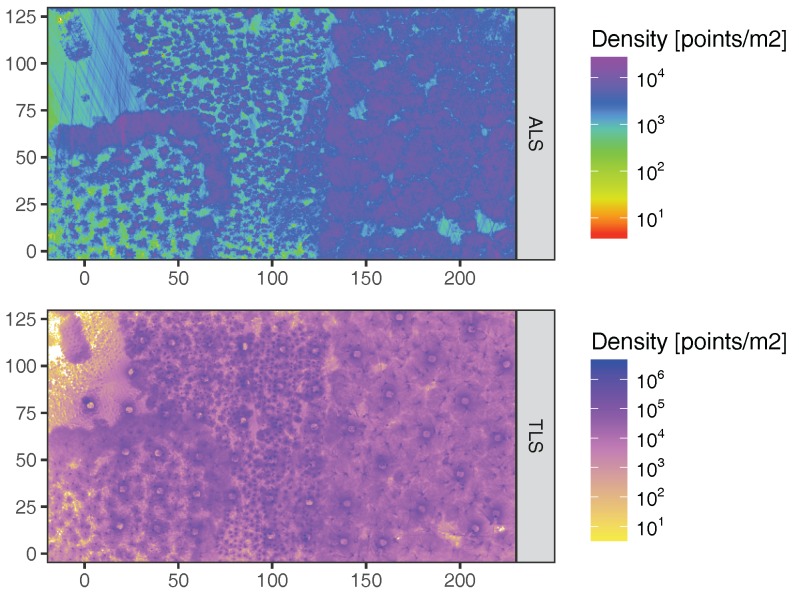
ALS and TLS point cloud density maps for whole study site at 0.5 m resolution in project coordinate system.

Figure 4 gives an overview of point density over the whole of the study site. While ALS point density was highest in tree crowns, visible as mushroom forms in the Old Beech and Oak area, TLS point density peaked at stem locations, visible as black specks in the TLS map. Furthermore, higher density areas were created by slight horizontal course corrections of the UAV, which are visible as stripe patterns in the density map, especially in the forest opening in the Northwest. Also more points were observed along the centre line of the plot in WE direction due to the higher overlap of flight lines in that area, i.e., northern and southern flight lines contribute to the centre locations. This can be seen when comparing Beech areas close to WE centre line and Beech in upper right of Figure 4, around [160,60] and [210,110], respectively. In case of TLS fewer points were registered around scan positions, which stems from the restriction in zenithal scanning angle of the VZ-400 scanner. Overall, ALS point density was about 2 orders of magnitude lower than TLS for the given scan configurations. It was on average 5344 $\mathrm{points}/m^{2}$, 3081 $\mathrm{points}/m^{2}$, 3005 $\mathrm{points}/m^{2}$, 2965 $\mathrm{points}/m^{2}$ and 3004 $\mathrm{points}/m^{2}$ for the Old Beech and Oak, Giant Fir, Norway Spruce, Young Beech, and Douglas Fir plots, respectively.

**Figure 5:**
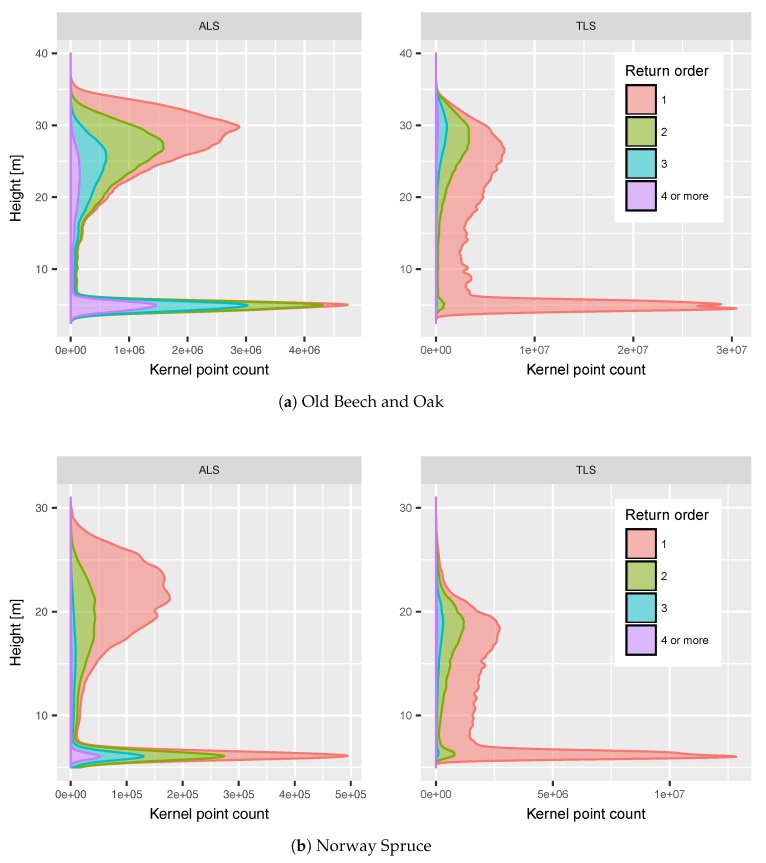
Vertical return density profiles smoothed with Gaussian kernel of all points in two areas of interest (see Figure 2), height reference is lowest point found in sub-area.

Figure 5 shows vertical return profiles of two focus areas representing different canopy architectures. While the Old Beech and Oak canopy had higher trees of up to 35 m with only few branches on the lower levels, the Norway Spruce canopy had trees up to 30 m and a considerable higher number of small, horizontally oriented branches below the crown level. The distribution of ALS and TLS points was similar for both canopies, but ALS hit relatively more often the upper canopy levels. This is clearly the effect of perspective of the ALS from above and TLS from below the canopy. Considering the distribution of return order in the upper canopy, the Old Beech and Oak canopy showed many more higher order returns than the Norway Spruce canopy. This could be explained by the foliage coverage of the Norway Spruce: while the water in the green needles allowed first order returns, it absorbed too much of the pulse energy to allow higher order return from within the clumped shoots. On the other hand, the not fully developed Old Beech and Oak canopy allowed partially intercepted beams and therefore multiple returns.

Despite the perspective of the ALS above the canopy, it hit the ground level in similar proportions as the TLS relative to all returns. This was clearly possible due to the multi-return capabilities of the VUX${}^{®}$-1UAV scanner. Returns up to 7th order could be recorded over the whole study area. This is in contrast to the TLS that is dominated by 1st order returns, which results from a higher proportion of hard targets like wood and ground elements. The ground returns were spread over some range of heights due to slightly sloped terrain and ground features like dead trees and hollows.

**Figure 6:**
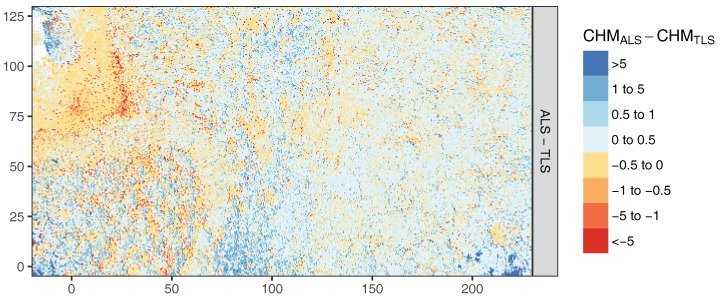
Differences in Canopy Height Model (CHMs). Colour height bins were chosen according to histogram and to facilitate evaluation of the sign of the difference.

Figure 6 shows the CHM difference map. The underlying DTM, DSM and CHM maps can be found in the supplementary material. General agreement between both maps was very high with a dominance of small offsets of ±0.5 m. However, an overall pattern can be observed similar to relieve shading resulting in positive differences in eastern and negative in western directions The pattern is not regular over the whole study area. For instance, it is strong over the Douglas Fir plot in the south-west and less pronounced over the Old Beech and Oak plot. This pattern stems from slight mis-registration of the point clouds of about 0.5 to 1 m at the crown level. One major source of error could have been crown movement by wind, a common problem in LiDAR field data acquisition [11]. This would also explain the pronounced pattern in the Douglas Fir plot with its high trees (up to 35 m) that are more susceptible to wind than the lower trees in the other plots. Finally, the TLS did not cover a small area in the Northwest that was occluded by a copse in the middle of the grass patch.

**Figure 7:**
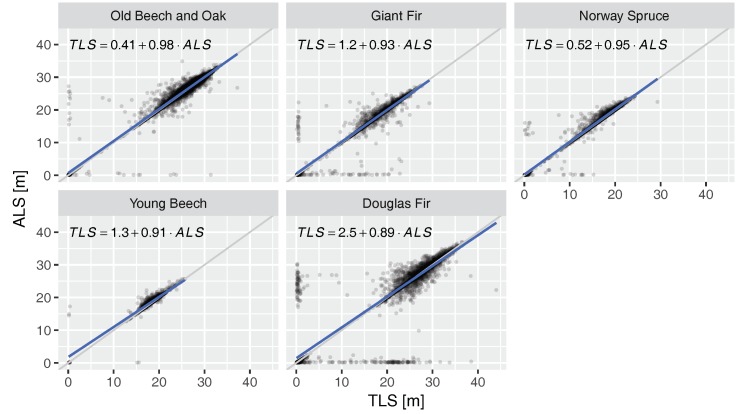
Differences in CHM per plot. Ordinary least squares regression lines in blue and formulas. Grey lines are 1:1 lines. Points are transparent to facilitate identification of high density clusters.

The mis-registration can also be found in the scatterplot of height differences in the plots in Figure 7: extreme cases can be found along the *x* and *y* axes. They represent cases when either the ALS or the TLS hit the crown and the other the ground. Outliers along the *x* and *y* axis represent mainly the western and eastern crowns sides, respectively. Nonetheless, all scatterplots confirm the high agreement of ALS and TLS. However, similar to the vertical profiles (Figure 5) also in the case of the CHMs ALS tended to detect higher points in the canopy, resulting in overall higher CHM. For instance, the ALS CHM was 6.1 cm and 12.2 cm higher for the Giant Fir and Old Beech and Oak plots, respectively. The difference for all cells over all plots was 11.5 cm.

**Figure 8:**
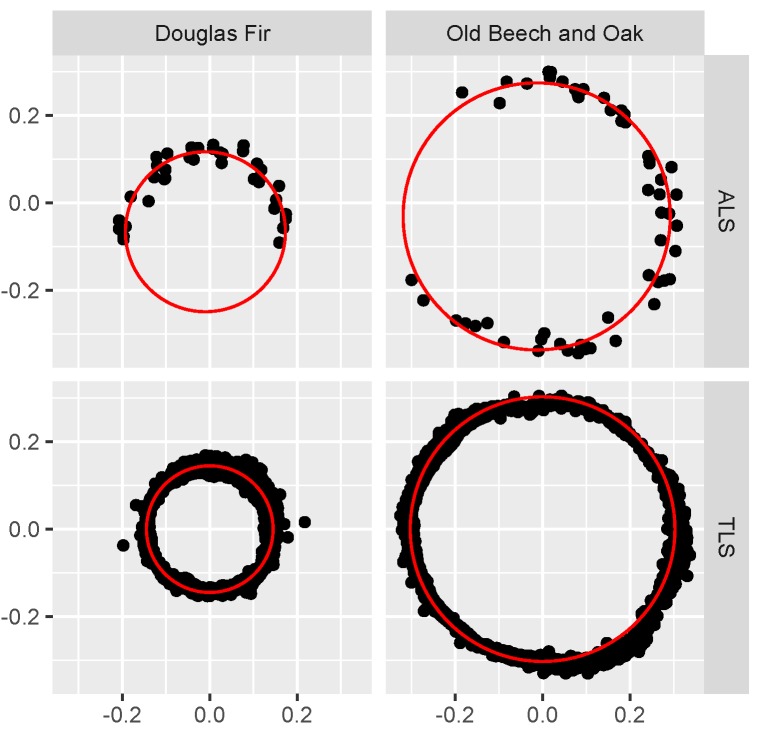
Samples of fitted circles to estimate DBH in the *x*-*y*-plane, *x* and *y* axis in cm.

**Figure 9:**
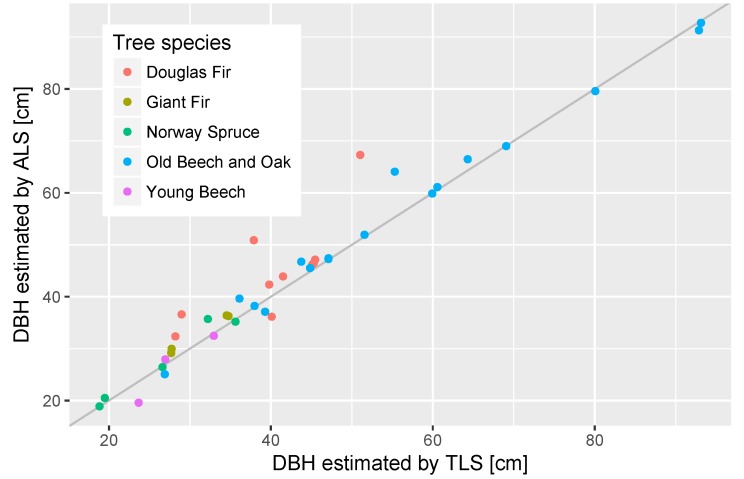
DBH of TLS compared to ALS. Grey line is 1:1.

Out of the 58 extracted tree stems, 39 were found to be suitable for DBH estimation. Of the accepted 12 trees stems had low level branch points that had to be removed to make the circle fitting possible. The 19 unsuitable trunks would clearly fail to form a circle (17) or had bifurcated trunks that violated the assumption of a single, circular trunk (2). The rejected cases were mainly found in the dense Giant Fir and Norway Spruce plots, and in the Young Beech plot with small overall trunks and branches on the lower canopy levels. Each ALS and TLS point ring contained 40 and 5522 points on average, respectively. Figure 8 shows examples of 2 trunks and the resulting fitted circles. In both cases there was a large number of TLS points available, while only few ALS points covered the stem at the DBH height and in the case of Douglas Fir it was also only from one direction. However, it was still possible to approximate the stems with circles. In Figure 9 the performance of ALS fitted circles in comparison to TLS fits can be seen. Both agree well with a correlation coefficient of 0.98 and root mean square error of 4.24 cm, while the range of estimated DBH was 19 to 93 cm. In comparison to TLS ALS estimates were 1.71 cm larger.

**Figure 10:**
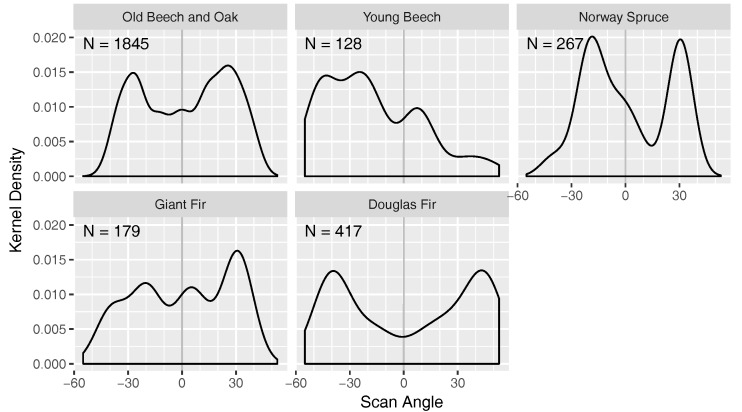
ALS scan angles under which the points considered for the DBH estimation were observed. N denotes the number of points for each plot.

Another interesting observation concerned the scan angles and return orders of the ALS points that were available for the DBH estimation. The scan or body across angle is the angle under which a laser pulse was shot from the scanner. It is 0° perpendicular to the rotor arms when mounted, i.e., a scan angle of 0° would describe nadir when the RiCOPTER flies perfectly horizontal. Large positive and negative scan angles result in low incidence angles. Figure 10 shows the distribution of scan angles for the sampled points that were used for the DBH estimation. The distributions were strongly bimodal for the Old Beech and Oak, Norway Spruce and Douglas Fir plots with peaks between −20° to −40° and 20° to 40°. The distribution of the Young Beech points were dominated by two trees that were hit from flight lines with the same orientation, resulting in a peak around −30°. Even though the RiCOPTER does not always fly perfectly horizontal, because it has to compensate the wind direction, generally scan angles around 30° to 40° seem to be well suited for detection of trunks. Probably self-occlusion of the observed tree and occlusion by other trees is minimal at these angles. Furthermore, trunk points were constituted of 14.6% 1st, 25.8% 2nd, 29.4% 3rd, and 30.2% 4th or higher return order. This underlines that the multi-return capability of the VUX${}^{®}$-1UAV was beneficial to observe trunks.

## 6. Discussion

Development of LiDAR technology and algorithms in recent years has shown great potential to support forestry practices, in particular geometric characterisation of plots up to unbiased estimation of AGB [4]. In this context TLS yields a data source of unprecedented detail and accuracy. However, TLS acquisition can be labour intense and time consuming especially in challenging environments like tropical forests [11]. New UAV-borne LiDAR technology can possibly accelerate these field campaigns and provide a larger coverage.

In this context, the RIEGL RiCOPTER with VUX${}^{®}$-1UAV has proven useful to characterise the Speulderbos forest plot. CHMs were successfully derived and showed good agreement with TLS. Canopy height estimated by the ALS were generally higher. This could be expected by its viewing perspective from above the canopy and the known shortcoming of TLS to not always detect the top of canopy (e.g., [23]). However, the difference of on average 11.5 cm falls within the precision of traditional field measurements for tree height of 50 cm [24]. Concerning the estimation of individual tree height, Wallace el al. [17] found good agreement with field measurements of 0.35 m (mean absolute error) by using point clouds of up to 300 $\mathrm{points}/m^{2}$ density.

Multi-return and side-looking capabilities proved to be important features of the VUX${}^{®}$-1UAV to scan trunks and estimate DBH for a number of trees under different canopy conditions (Figure 9). While other UAV LiDAR systems are also able to record multiple targets per pulse, not many systems are able to acquire data under larger scan angles (>30° off nadir). Nonetheless, a sufficient number of points could not be collected for all sampled trunks, mainly in the dense, narrow spaced needle-leaf plots. Repeated flights over the same plots with varying flight paths could result in better coverage. The average root mean square error of 4.24 cm between TLS and ALS is comparable to reported deviations of TLS and traditional methods of 0.7 to 7.0 cm (RMSE) [1]. However, the DBH estimation error is still much larger than the precision of traditional methods of ~0.3 cm [24].

The scan angles that proved optimal to scan the trunk samples (Figure 10) have implications for the flight preparation. The targeted plots should be always well covered, possibly with flight lines that overshoot the plot area. For instance if the flight height is at 90 m and the optimal angle is assumed to be 30°, the flight trajectory should overshoot by ~52 m. However, it is difficult to say how general the optimal scan angles found in this study are. In any way, we found that multiple flight lines, made possible through the long air-borne time, were contributing to a better sampling from different directions. In this respect more lines at faster speed, should be preferred to fewer at lower speed assuming same airborne time. Maximising line crossings and multiple flights should be considered as well. The later will be primarily restricted by the number of battery packs available.

Initial point cloud production of VUX${}^{®}$-1UAV data solely relies on the on-board IMU and GNSS post processing. This is possible because of the IMU’s accuracy, which on the other hand results in a large weight for the scanner. This drives the weight of the whole system, since heavier sensors also require larger payload capacity and thus larger UAVs. Together with the system requirement of long endurance this brought the total system weight up to just under the legal limit for mini-UAV (personal communication RIEGL, 2016). This design decision makes it unnecessary to employ algorithms that reconstruct the trajectory in the post-processing as shown in previous studies [16,18]. However, it makes the availability of GNSS base station data a hard requirement that might be difficult to fulfil in remote areas. Also the system weight can be a logistical challenge in such cases.

Even though this study did not aim to conduct full plot inventories, the data shows promising attributes to extend the analysis in that direction. One important step for this would be to detect single trees in all plots. Wallace et al. [25] produced detection rates of up to 98% with point clouds of 50 $\mathrm{points}/m^{2}$ density. Therefore, detection should be achievable with the ~3000 $\mathrm{points}/m^{2}$ RiCOPTER point clouds. Based on the detected trees, single tree height can be estimated. However, traditional forest inventory data would be necessary for validation.

Apart from characterising traditional forest metrics, UAV-borne LiDAR could also be utilised as a flexible, higher resolution alternative to manned airborne LiDAR, especially to study foliage. In that case several published algorithms could be employed [26,27,28,29] and tested if they are applicable on higher density point clouds. Moreover, reliable and mobile systems like the RiCOPTER are suitable for multi-temporal studies [15].

## 7. Conclusions

This study presented first results and experiences with the RIEGL RiCOPTER with VUX${}^{®}$-1UAV ALS with its components and processing work flow, and its performance in estimating CHM and DBH compared to TLS. As first steps we compared the RiCOPTER with the well tested RIEGL VZ-400 TLS by deriving CHM and estimating DBH. CHMs showed only small differences that could be explained by the perspective of the RiCOPTER above the canopy, resulting in different vertical detection profiles that facilitate the discovery of highest points in the canopy, which is not always possible with TLS. Additionally, the multi-return and side-looking capabilities of the VUX${}^{®}$-1UAV scanner proved beneficial to detect trunk elements. This feature will be valuable when more sophisticated 3D modelling is to be applied. However, not all sampled tree trunks were sufficiently covered with points, so that more flights or different flight patterns are necessary to achieve better coverage. Overall, the RiCOPTER could produce comparable results to the VZ-400. Further experiments should be directed to test explicit quantitative structural modelling to derive AGB from the RiCOPTER point clouds as well as co-registration strategies of multiple flights and with TLS systems.

## Figures and Tables

**Table 1:** VZ-400 and VUX${}^{®}$-1UAV main characteristics.

Characteristic	VZ-400 ^1^	VUX-1UAV ^2^
Maximum Pulse Repition Rate (PRR) (kHz)	300	550
Maximum effective measurement rate (kHz)	120	500
Minimum|Maximum range (m)	1.5|350 ^3^	3|920 ^4^
Accuracy|Precision (mm)	5|3	10|5
Laser wavelength (nm)	1550	1550
Beam divergence (mrad)	0.3	0.5
Weight (kg) ^5^	9.6	3.75

^1^ high speed mode, incl. online waveform processing; ^2^ 550 kHz mode; ^3^ at target ρ≥0.9; ^4^ at target ρ≥0.6; ^5^ without battery and tilt mount.

## Supplementary Materials

The following are available online at http://www.mdpi.com/1424-8220/17/10/2371/s1, Figure S1: Digital Elevation Models at 0.5 m resolution in project coordinate system, blank cells did not contain points; Figure S2: Digital Surface Models at 0.5 m resolution in project coordinate system, blank cells did not contain points; Figure S3: Canopy Height Models at 0.5 m resolution in project coordinate system, blank cells did not contain points.
