# Supplementary material for: Comparing RIEGL RiCOPTER UAV LiDAR Derived Canopy Height and DBH with Terrestrial LiDAR
Source: Sensors (Basel). 2017 Oct 17;17(10):2371. doi: 10.3390/s17102371 (PMC5677400; doi:10.3390/s17102371)
Supplement: Supplementary file 1 [file sensors-17-02371-s001.pdf]

Supplementary Materials: Comparing RIEGL RiCOPTER UAV LiDAR Derived Canopy Height and DBH with Terrestrial LiDAR

Benjamin Brede [1,*], Alvaro Lau [1,2], Harm M. Bartholomeus [1] and Lammert Kooistra [1]

[1] Laboratory of Geo-Information Science and Remote Sensing, Wageningen University & Research, Droevendaalsesteeg, 36708 PBWageningen, The Netherlands; alvaro.lausarmiento@wur.nl (A.L.); harm.bartholomeus@wur.nl (H.M.B.); lammert.kooistra@wur.nl (L.K.)

[2] Center for International Forestry Research (CIFOR), Situ Gede, Sindang Barang, Bogor 16680, Indonesia

* Correspondence: benjamin.brede@wur.nl

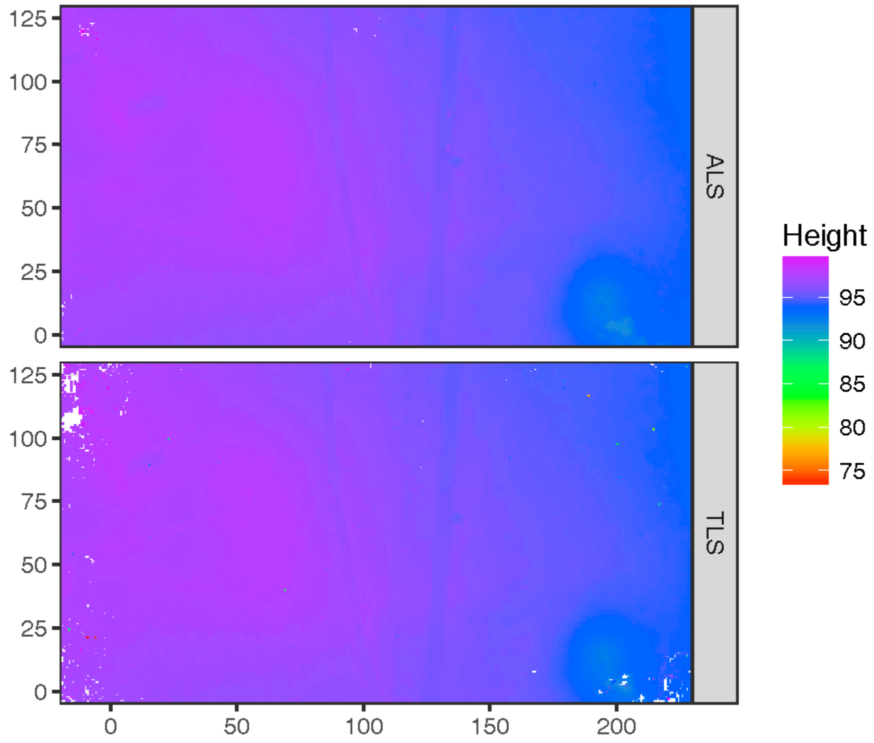

Figure S1. Digital Elevation Models at 0.5 m resolution in project coordinate system, blank cells did not contain points.

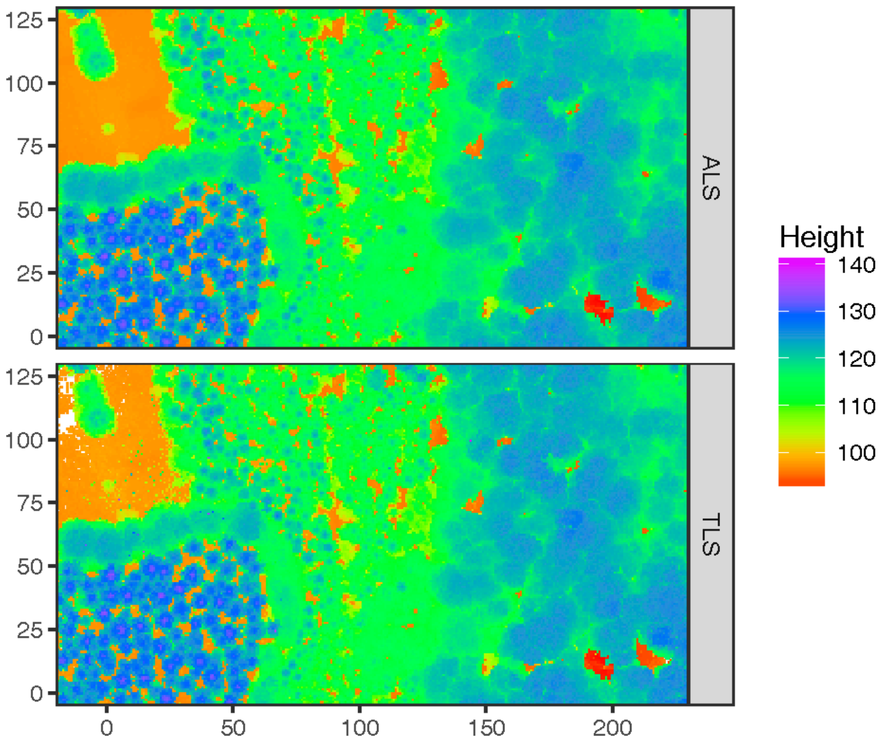

Figure S2. Digital Surface Models at 0.5 m resolution in project coordinate system, blank cells did not contain points.

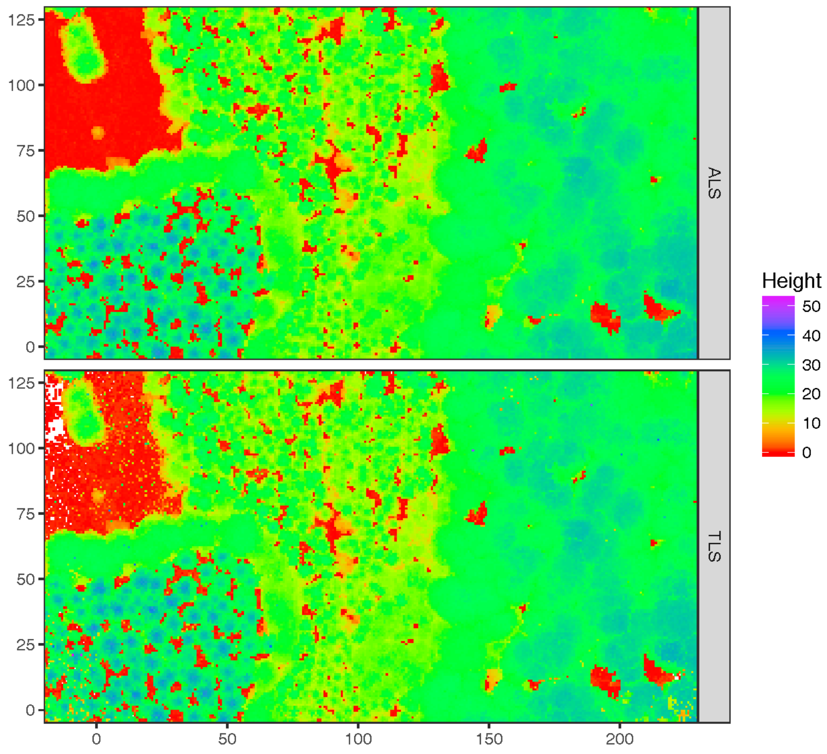

Figure S3. Canopy Height Models at 0.5 m resolution in project coordinate system, blank cells did not contain points.
